# The impact of additional visual tasks in physical exercise on balance ability among 9–10-year-old children: the mediating effect of visual acuity

**DOI:** 10.3389/fpubh.2023.1270947

**Published:** 2024-01-08

**Authors:** Rongbin Yin, Guiming Zhu, Anqi Liu, Miyu Wang, Liangtao Li, Shengting Dai

**Affiliations:** ^1^School of Physical Education, Soochow University, Suzhou, China; ^2^Department of Physical Education, Suzhou Vocational University, Suzhou, China; ^3^School of Sports Science and Engineering, East China University of Science and Technology, Shanghai, China

**Keywords:** children, visual tasks, physical exercise, visual acuity, balance ability

## Abstract

**Purpose:**

This study aimed to explore the effects of additional visual tasks in physical exercise on the vision and balance ability of children, and to verify whether children’s vision mediated the influence of physical exercise on their balance ability.

**Methods:**

The study randomly selected 86 students aged 9–10 years old from a school in Suzhou city, dividing them into an experimental group (*n* = 43) and a control group (*n* = 43). The experimental group participated in physical exercise with additional visual tasks, while the control group engaged in routine physical exercise. The experiment lasted for 16 weeks, with kinetic visual acuity (KVA), uncorrected distance visual acuity (UDVA), static balance, and dynamic balance measured before and after the experiment.

**Results:**

The results showed that after the experiment, the experimental group had significantly improved kinetic visual acuity (KVA), uncorrected distance visual acuity (UDVA), static balance, and dynamic balance. In contrast, the control group had significantly decreased kinetic visual acuity, no significant improvement in uncorrected distance visual acuity, and no significant difference in dynamic balance and static balance. In the experimental group, there was a moderate positive correlation between kinetic visual acuity and uncorrected distance visual acuity, and a moderate positive correlation between uncorrected distance visual acuity and both static and dynamic balance. The study also found that uncorrected distance visual acuity partially mediated the effect of additional visual tasks during physical exercise on static and dynamic balance among children.

**Conclusion:**

In conclusion, adding visual tasks to physical exercise had a positive effect on improving children’s vision and balance ability. Kinetic visual acuity and uncorrected distance visual acuity were positively correlated, and uncorrected distance visual acuity was positively correlated with both static and dynamic balance. Uncorrected distance visual acuity partially mediated the effect of physical exercise on children’s balance ability.

## 1 Introduction

In the primary school stage, the physical and psychological development of students are immature, and the ability to control the body is poor. Therefore, in daily life, children often experience phenomena such as ankle sprains, instability while standing, and easy falls. These physical symptoms indicate children’s poor balance function. Balance is the ability to maintain the body’s center of gravity on a support base (1), which is a prerequisite for normal physical development among children (2). Balance ability is divided into two forms, one is the ability to maintain a specific posture of the body in a static state, that is, static balance. The other refers to the ability of people to regulate their body posture during exercise, that is, dynamic balance. Developing children’s balance ability can not only maintain body posture but also reduce the risk of sports injuries. Therefore, as an important part of children’s physical fitness (3), if its development is hindered in some extent in the early stage of children, it may negatively affect children’s learning and mastering sports skills, or even their participation in sports activities (4). KVA (Kinetic vision acuity) refers to the visual ability of the human eye to observe the forward and backward direction of an object. Primary school is a key period of human growth and development, and also a critical period for the development of kinetic visual acuity (KVA) and balance ability. Children’s KVA and balance ability will gradually improve with age (5, 6), and 8–14 years old is a sensitive period for the development of KVA (7), while before the age of 10 is the critical period for the development of balance ability (8). Therefore, scientific training methods should be adopted in the childhood stage to enhance children’s KVA and balance ability, and to promote the overall improvement of their physical health.

Physical exercise is an important means to improve children’s balance ability, and also an effective way to improve their eyesight. Chen et al. (9) found that regular physical exercise can effectively improve the proprioceptive agility of students’ muscles and the stability of the vestibular system, and increase the ability to adjust when the body posture changes. Park et al. (10) and others found that regular physical exercise could improve children’s balance ability, enhance their ability to control body changes, thus reducing the incidence of sports injuries. And the physical exercise of different sports has different effects on improving children’s balance ability. Liu (11) found that skipping exercise has a positive effect on enhancing the balance ability of primary school students, and significantly improves the proprioception and static and dynamic balance ability of lower limbs through 12 weeks of skipping exercise. Li et al. (12) football can effectively improve students’ lower limb muscle strength and ankle proprioception, so as to achieve better balance improvement. In addition, physical exercise can replace the function regulation training, enhance the accuracy and coordination of the ciliary muscle regulation, promote changes in eye parameters, and promote visual health (13, 14).

Physical exercise can improve vision by enhancing the far-near visual accommodation ability through visual recognition tasks, relieving the tension of the ciliary muscle, and promoting the improvement of kinetic visual acuity (15). Uncorrected vision refers to the measured vision that has not been corrected by any optical lens. As kinetic visual acuity (KVA) is significantly positively correlated with uncorrected distance visual acuity (UDVA), improving KVA is beneficial to improving UDVA (16). Therefore, specially designed physical exercise can improve children’s eyesight, promote their visual health development, and strengthen their visual function.

When people are doing exercise, their eyes perceive external environment and transmit information to the brain that receives feedback from proprioceptors and vision, and controls eyes to maintain clear vision, thus maintaining appropriate body postures (17). Studies have also shown that visual limitation significantly affected posture stability. When the body moved, it was more easily to maintain balance if eyes could see the position of the body (18). Bednarczuk et al. (19) believed that people with visual impairment received poor visual stimulation, compared with people with typically-sighted, which affected their static balance. Huang (20) found that students with visual impairments result in limited physical activity, which affects the development of balance, which is especially important for students with low vision. Liu et al. (21) found that vision and visual accommodation are important factors in maintaining balance in children, and that children with visual disabilities have lower balance than typically-sighted children. Song et al. (22) found that visual acuity was essential for physical activity and postural control, and people with visual impairment had worse balance ability than typically-sighted individuals. Singh et al. (23) have shown that vision, specific postures in sports, frequency and duration of exercise played decisive role in effects of physical activity on standing postural stability. Onofrei and Amaricai (24) pointed out that physical activity and vision were important factors affecting the postural balance of healthy students, and body balance was affected by the interaction of physical activity and vision. To sum up, children’s visual acuity had a certain impact on their balance ability. With visual impairment, accuracy of individual’s body movement obviously reduced.

Currently, Chinese and foreign research has found that physical exercise can promote the development of children’s visual health and balance ability, but the mediating role of children’s eyesight in the impact of physical exercise on balance ability has not been thoroughly studied. Therefore, based on the above theoretical basis, the hypothesis is proposed that children’s vision has a mediating effect on the impact of physical exercise on balance ability, and this hypothesis will be verified through experimental research.

## 2 Materials and methods

### 2.1 Participants

86 children, aged 9–10, were randomly selected from two 4th grade classes in a primary school in Suzhou city as participants. They were divided into an experimental group and a control group, with each group being assigned randomly by class. The experimental study was conducted in the Physical Education and Health programme. The experimental group emphasized additional visual training during their physical exercise, while the control group followed a regular physical exercise routine. There were no differences in KVA, UDVA, static balance, and dynamic balance between the two groups, making them suitable for the experiment. Specific information about the participants can be found in Table 1.

**Table 1:** Participants demographic characteristics (*N* = 86, M ± SD).

Group	Experiment group	Control group	Value of *p*
Total	43	43	
KVA	0.409 ± 0.19	0.414 ± 0.25	0.923
UDVA	4.705 ± 0.30	4.744 ± 0.36	0.584
Static balance	30.19 ± 10.30	29.87 ± 8.54	0.878
Dynamic balance	84.49 ± 8.76	84.24 ± 7.58	0.891

Participant selection criteria were as follows: (1) no orthokeratology lenses; (2) no pathological eye diseases; and (3) normal cognitive and motor functions. The study protocol was in accordance with the Helsinki Declaration and was approved by the Ethics Committee of Suzhou University (No. SU-DA20201010H01).

### 2.2 Experimental plan

The intervention plan for additional visual tasks will be integrated into the teaching of ball sports. During the teaching process, quantitative static and dynamic visual targets will be added to increase students’ training frequency of observing distant and near visual targets, and to promote sufficient adjustment training of the ciliary muscle. When using ball sports as a carrier for adjusting the ciliary muscle, visual target observation for 3 s and the 30-frequency distance-near switch will be selected as the baseline for ciliary-muscle training. The experimental group will perform additional visual task exercises (ciliary-muscle adjustment training) during physical exercise, while the control group will perform regular physical exercise.

Children aged 9–10 are in a critical period of motor development and a sensitive period of physical development. In addition, the fourth grade of primary school is also an important stage to develop balance skills. The experimental content will be selected based on the physical and mental development characteristics of 9–10-year-old children, combined with the standards of physical education and health courses, and school curriculum tasks, whereby basketball and football are selected for related experiments. Basketball and football programs each have 8 weeks of study. In the first 8 weeks of the experiment, I will learn basketball sports, mainly including: high dribble, low dribble, two-handed chest pass and catch, two-handed chest shot and pass and cut tactics (Table 2). 8 weeks after the experiment, I will learn football sports, mainly including: instep inside dribble, instep front dribble, inside foot pass and catch, inside foot kick and pass and cut coordination tactics. In addition to the additional visual adjustment training tasks, the groups will be unified in terms of the sports venue, exercise time, and exercise project to ensure the rigor of the experiment. The intervention period for the experiment is 16 weeks, with three interventions per week, each lasting for 40 min. In the course of the experiment, in order to avoid the influence of irrelevant variables on the experimental research, to ensure the orderly conduct of the experiment. From the beginning to the end of the experiment, the physical education of the experimental group and the control group were all taught by myself to ensure the uniformity of the teaching teacher, exercise content, exercise load, exercise density and venue equipment, and in strict accordance with the teaching plan in accordance with the requirements of the physical education and health curriculum standards.

**Table 2:** Experiment content.

Classification	Projects	Exercise content	Exercise time	Movement frequency	Duration of ciliary muscle intervention
Open Motor skills	Basketball	High dribbling, low dribbling, two-handed chest pass, two-handed chest shot, passing and cutting tactics, etc.	Total 4 weeks 40 min each time	Three times a week	Control group without ciliary muscle intervention and normal participation in physical education classes. The hourly value of the ciliary muscle intervention in the experimental group was 3 s/visit, and the distance-approach vision was 30 frequencies; since the ciliary muscle intervention is an exercise, it cannot be estimated accurately and depends on the content of the exercise.
	Soccer	Dribbling on the inside of the back of the foot, dribbling on the front of the back of the foot, passing and receiving on the inside of the foot, kicking on the inside of the foot, passing and cutting tactics, etc.	Total 4 weeks 40 min each time	Three times a week	

The design of additional visual tasks includes three aspects. (1) Based on the learning characteristics of 9–10-year-old children, the content will be arranged from easy to difficult, and from simple to complex. In the process of experimental intervention, according to the order, stages and differences of students’ physical and mental development, the experimental process was arranged according to the teaching concept of “learning, practice, competition and evaluation,” and the physical exercise of additional visual training tasks was integrated into the preparation part, the basic part and the end part of class teaching. (2) The formation process of motor skills can be divided into three interrelated stages: generalization, differentiation and consolidation. The development stage of motor skills is called the action automation stage (17). It conforms to the laws of sports skill learning and follows the rules of sports skill formation. (3) It conforms to the essential characteristics of physical exercise, which is to enhance individual’s physical fitness and promote health. In accordance with the requirements of the compulsory education physical education and health curriculum standards, the average heart rate and practice density of each class meet the requirements.

### 2.3 Testing methods

Before the experiment (week 1) and after the experiment (week 16), subjects’ KVA, UDVA, static balance, and dynamic balance in each group will be measured. The test place was in a classroom of an experimental primary school in Suzhou City, which was quiet and full of light. To ensure the accuracy and effectiveness of the experimental data, the testing procedures are strictly followed according to the standards, and the measurement and recording of each data item will be performed by the same person before and after the experiment. During the test, the students lined up in order according to their student numbers, kept quiet, and took the eyesight and balance tests in turn.

#### 2.3.1 KVA test

The KVA test was conducted using a kinetic visual acuity testing device (XP.14-TD-J905 model). The detection range value was between 0.1–1.6, with higher values indicating better KVA. The subject is asked to sit in the designated position in front of the instrument, remain upright, and look inward with both eyes close to the visual hole. After the student is ready, the tester swips the card and clicks to start. A simulated “C” type visual beacon approaching itself from 50 meters away appears in the instrument. The notch direction of the “C” type visual beacon is divided into up, down, left and right, and the simulated approach speed is 30 km/h. The subjects held the rocker with their hands, saw the “C” notch direction and quickly pushed it in that direction. Each student underwent three consecutive tests, and the average of the three tests results was taken as the final value of the student’s KVA.

#### 2.3.2 UDVA test

The “Standard Logarithmic Vision Chart” was used to test UDVA, strictly following the standard testing method and procedure. The subject stood 3 meters away from the visual acuity chart and gently covered one eye with an eye shield. Start with the maximum visual icon of the first line (4.0 line visual icon) with an indicator bar, top to bottom, and check line by line, requiring the subject to speak or indicate the notch direction of the visual icon within 3 s, and the visual acuity represented by the last line visual icon that the subject can see is the visual acuity of the subject’s eye. The standard of each line is to measure the smallest line of sight that can be recognized by the eye (the number of correct sight signs should be more than half of the total number of the line of sight signs), and write down the visual record value of the line of sight, that is, the vision of the eye. If the subject wore glasses, they were removed before testing. The final value was taken from the student’s right eye as the UDVA test value.

#### 2.3.3 Static balance test

Static balance was tested using the “Open-Eyes and Single-Leg Standing Test.” The subject stood in front of the tester and, when the tester announced “testing begin,” the subject opened their eyes, put their hands on their hips, stood on their dominant foot (barefoot), lifted their non-dominant leg, bent their knee, kept a gap between their legs, and was not allowed to borrow support from one another. Timing stopped when the non-dominant leg touched the ground or when the body strongly swayed during standing. The subject was tested three times, each test was followed by a 1 min rest, and the next test was performed. The average of the three tests results was taken as the final value of the subject’s static balance.

#### 2.3.4 Dynamic balance test

The Y-Balance Test instrument was used to test dynamic balance. Before testing, the subject’s leg length was measured with the subject’s body kept upright and the lower limbs extended, measuring the distance from the anterior superior iliac spine to the lower edge of the ankle bone on the non-dominant side. During testing, the subject was required to support themselves on the dominant leg while extending their non-dominant leg in three directions: forward, back inward, and back outward. Meanwhile, the subjects were required to remain upright, with their knees not bent and their feet naked. The evaluation standard after testing was: (the sum of the distance extended in three directions)/(3 times leg length) × 100%, with a larger ratio indicating better dynamic balance. During the test, the non-supporting foot in the forward side direction as the starting point, clockwise to complete the test in three directions. After pushing the caliper to the furthest distance in each direction, the leg should be withdrawn to return to the starting point, and then the next direction should be tested. If the foot touches the ground during the test, the direction should be re-measured. In each direction, the non-supporting foot pushed the caliper to the farthest end and held it for 1 s, and the distance was noted. Each direction was tested three times, and the average of the three tests results was taken as the final value for that direction.

### 2.4 Statistical analysis

Data entry and organization were performed using Excel 2019 software, and the experimental data was statistically analyzed using SPSS 25.0, the statistical software. The data of KVA, UDVA, static balance, and dynamic balance all met the homogeneity test, normal distribution, and equal variance. Data results were presented as mean ± standard deviation (M ± SD). 2 × 2 mixed design ANOVA and independent sample *t*-tests were used to analyze the testing data of KVA, UDVA, static balance, and dynamic balance of the subjects. Pearson correlation analysis and regression analysis were used to test the mediating effect of visual acuity on the subjects.

## 3 Results

### 3.1 Effects of additional visual tasks in physical exercise on children’s visual acuity and balance ability readings

To investigate the effects of additional visual tasks in physical exercise on children’s visual acuity and balance abilities, 2 × 2 mixed ANOVA were conducted for KVA, UDVA, static balance, and dynamic balance. Changes in students’ visual acuity and balance abilities were observed as a result.

2 (Sessions: pre-test, post-test) × 2 (Groups: experimental, control) mixed ANOVA were conducted on visual acuity and balance ability (Tables 3, 4). 2 (Session: pre-test, post-test) × 2 (Group: experimental, control) mixed ANOVA was conducted on KVA. The results showed the non-significant main effect of session (*F*(1,84) = 3.401, *p* > 0.05), the significant main effect of group (*F*(1,84) = 14.310, *p* < 0.05), and the significant two-factor interaction effect (*F*(1,84) = 37.585, *p* < 0.05). Simple effects analysis showed that in the experimental group, there was the significant difference in KVA before and after the experiment (*F*(1,84) = 31.799, *p* < 0.05); in the control group, there was the significant difference in KVA before and after the experiment (*F*(1,84) = 9.187, *p* < 0.05). 2 (Session: pre-test, post-test) × 2 (Group: experimental, control) mixed ANOVA was conducted on UVDA. The results showed that the significant main effect of session (*F*(1,84) = 17.407, *p* < 0.05), the non-significant main effect of group (*F*(1,84) = 3.119, *p* > 0.05), and the significant two-factor interaction effect (*F*(1,84) = 43.750, *p* < 0.05). Simple effects analysis showed that in the experimental group, there was the significant difference in UVDA before and after the experiment (*F*(1,84) = 58.175, *p* < 0.05); and in the control group, there was no significant difference in UVDA before and after the experiment (*F*(1,84) = 2.982, *p* > 0.05). 2 (Session: pre-test, post-test) × 2 (Group: experimental, control) mixed ANOVA was conducted on static balance. The results showed the significant main effect of session (*F*(1,84) = 38.119, *p* < 0.05), the significant main effect of group (*F*(1,84) = 4.211, *p* < 0.05), and the significant two-factor interaction effect (*F*(1,84) = 14.600, *p* < 0.05). Simple effects analysis showed that in the experimental group, there was the significant difference in the static balance before and after the experiment (*F*(1,84) = 49.950, *p* < 0.05), and in the control group, there was no significant difference in the static balance before and after the experiment (*F*(1,84) = 2.769, *p* > 0.05)0.2 (Session: pre-test, post-test) × 2 (Group: experimental, control) mixed ANOVA was conducted on dynamic balance. The results showed the significant main effect of session (*F*(1,84) = 30.461, *p* < 0.05), the significant main effect of group (*F*(1,84) = 6.566, *p* < 0.05), and the significant two-factor interaction effect (*F*(1,84) = 21.997, *p* < 0.05). Simple effects analysis showed that in the experimental group, there was the significant difference in the dynamic balance before and after the experiment (*F*(1,84) = 52.115, *p* < 0.05), and in the control group, there was no significant difference in the dynamic balance before and after the experiment (*F*(1,84) = 0.344, *p* > 0.05). Further independent sample *t*-tests were conducted to analyze the differences in KVA, UDVA, static balance, and dynamic balance between the experimental and control groups, observing the changes in visual acuity and balance ability between them.

**Table 3:** Simple effects test results for balance ability.

Group	Balance ability	(I)	(J)	Mean difference (I–J)	Standard error	Value of *p*	Lower limit	Upper limit
Experimental group	Static balance	Pre-test	Post-test	−8.851	1.252	0.000	−11.341	−6.360
	Dynamic balance	Pre-test	Post-test	−7.552	1.046	0.000	−9.632	−5.472
Control group	Static balance	Pre-test	Post-test	−2.084	1.252	0.100	−4.574	0.407
	Dynamic balance	Pre-test	Post-test	−0.613	1.046	0.559	−2.694	1.467

* Indicates significant difference (*p* < 0.05).

**Table 4:** Simple effects test results for visual acuity.

Group	Visual acuity	(I)	(J)	Mean difference (I–J)	Standard error	Value of *p*	Lower limit	Upper limit
Experimental group	KVA	Pre-test	Post-test	−0.186	0.033	0.000	−0.252	−0.120
	UDVA	Pre-test	Post-test	−0.247	0.032	0.000	−0.311	−0.182
Control group	KVA	Pre-test	Post-test	−2.084	0.033	0.003	0.034	0.166
	UDVA	Pre-test	Post-test	0.056	0.032	0.088	−0.008	0.120

* Indicates significant difference (*p* < 0.05).

After conducting an independent sample *t*-test on test data of the KVA, UDVA, static balance, and dynamic balance in the two groups after the experiment (Table 5), the results showed significant differences (*p* < 0.05) in KVA, UDVA, static balance, and dynamic balance. The mean values of KVA, UDVA, static balance, and dynamic balance in the experimental group were higher than those in the control group.

**Table 5:** Difference analysis of visual acuity and balance ability in each group after the experiment (*N* = 86).

Indicators	Experiment group	Control group	Mean difference	Value of *t*	Value of *p*
KVA	0.595 ± 0.20	0.314 ± 0.15	0.28	7.373*	0.000
UDVA	4.951 ± 0.21	4.688 ± 0.35	0.26	4.224*	0.000
Static balance	39.04 ± 9.54	31.95 ± 8.73	7.08	3.593*	0.001
Dynamic balance	92.04 ± 6.26	84.86 ± 7.36	7.18	4.874*	0.000

* Indicates significant difference (*p* < 0.05).

### 3.2 Correlation analysis between visual acuity and balance ability of experimental group students

To explore the correlation between visual acuity and balance ability of the experimental group students, Pearson correlation analysis was conducted on their visual acuity and balance abilities. As shown in Table 6, there was a significant moderate positive correlation between KVA and UDVA in the experimental group students. However, there was no significant correlation between KVA and static balance or dynamic balance. There was a significant moderate positive correlation between UDVA and both static and dynamic balance in the experimental group students.

**Table 6:** Correlation coefficient of visual acuity and balance ability of students in the experimental group (*N* = 43).

Indicators	KVA	UDVA	Static balance	Dynamic balance
KVA	1	0.413**	0.028	0.087
UDVA		1	0.355**	0.480**
Static balance			1	0.086
Dynamic balance				1

** Indicates *p* < 0.01.

### 3.3 Mediation model test of the influence of physical exercise on the balance ability of fourth-grade students

Based on the research results of the correlation between physical exercise, UDVA, and balance ability, the mediation effect test process was used to test the mediation model of the influence of physical exercise on the balance ability of fourth-grade students. The hypothesis model of this study was divided into three analysis steps: (1) test coefficient c, conduct linear regression analysis on the two variables, with physical exercise with additional visual tasks as the independent variable (predictor variable) and balance ability as the dependent variable (criterion variable); (2) test coefficient a, conduct linear regression analysis on the two variables, with physical exercise with additional visual tasks as the independent variable and UDVA as the dependent variable; (3) test coefficient b, conduct linear regression analysis on the two variables, with physical exercise with additional visual tasks and UDVA as the independent variables and balance ability as the dependent variable. It was hypothesized that UDVA (M) was the mediator variable between physical exercise (X) and balance ability (Y), and the mediation effect of UDVA between physical exercise and balance ability was tested according to the mediation variable test procedure.

#### 3.3.1 Regression analysis of physical exercise and static balance

The dependent variable (criterion variable) of this study was static balance, and the independent variable (predictor variable) was physical exercise with additional visual tasks. Linear regression analysis was conducted on the two variables. From the regression analysis of the physical exercise with additional visual tasks and static balance, it showed *F* = 17.101, *p* = 0.000, R = 0.411, *R*^2^ change = 0.159, indicating that the regression equation had significant differences. The standardized regression coefficient of physical exercise with additional visual tasks on static balance of fourth-grade students was 0.411, and *p* < 0.05, indicating a significant difference.

#### 3.3.2 Regression analysis of physical exercise and dynamic balance

The dependent variable (criterion variable) of this study was dynamic balance, and the independent variable (predictor variable) was physical exercise with additional visual tasks. Linear regression analysis was conducted on the two variables. From the regression analysis of physical exercise with additional visual tasks and dynamic balance, it showed *F* = 21.173, *p* = 0.000, R = 0.449, *R*^2^ change = 0.192, indicating that the regression equation had significant differences. The standardized regression coefficient of physical exercise on dynamic balance of fourth-grade students was 0.449, and *p* < 0.05, indicating a significant difference.

#### 3.3.3 Regression analysis of physical exercise and UDVA

The dependent variable (criterion variable) for this study was UDVA, and the independent variable (predictor variable) was physical exercise with additional visual tasks. A linear regression analysis was conducted between the two variables. The regression analysis of physical exercise with additional visual tasks and UDVA yielded *F* = 19.300, *p* = 0.000, R = 0.432, *R*^2^ change = 0.177, indicating a significant difference in the regression equation. The standardized regression coefficient of physical exercise on UDVA for fourth-grade students was 0.432, with *p* < 0.05, showing a significant difference.

#### 3.3.4 Regression analysis of physical exercise, UDVA, and static balance

Through the study, it was found that physical exercise with additional visual tasks had a significant effect on the static balance of fourth-grade students. Therefore, a linear regression analysis was conducted between physical exercise with additional visual tasks and UDVA (together as predictor variable), and static balance (criterion variable). The regression analysis of physical exercise, UDVA and static balance yielded that *F* = 12.861, *p* = 0.000, R = 0.486, *R*^2^ change = 0.218, indicating a significant difference in the regression equation. The standardized regression coefficient of UDVA and static balance was 0.288, with *p* < 0.05, showing a significant difference.

**Figure 1:**
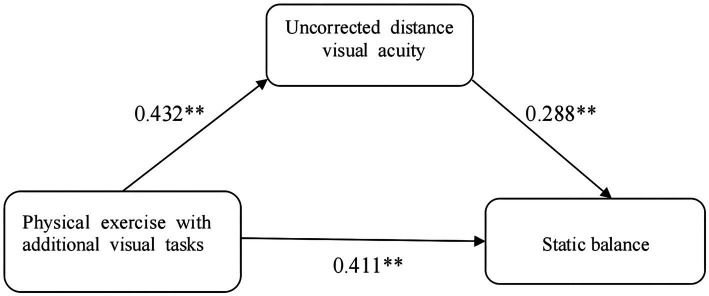
The mediating effect model of uncorrected distance visual acuity in physical exercise and static balance. The first step of the independent variable is physical exercise for additional visual tasks (X), the dependent variable is static balance (eyes open) (Y), and the regression analysis between the two variables is performed, which results in a standardized regression coefficient A1=0.411, *p*<0.05, *R*^2^ change=0.159; the second step of the independent variable is physical exercise for additional visual tasks (X), and the dependent variable is UDVA (M), and the two variables are subjected to a regression analysis resulted in a standardized regression coefficient A2 = 0.432, *p*< 0.05, *R*^2^ change = 0.177; the third step of the independent variable was physical exercise with additional visual tasks (X) and UDVA (M), and the dependent variable was static equilibrium (with eyes open) (Y), and a regression analysis was performed between the two variables, which resulted in a standardized regression coefficient A3 = 0.287, *p* < 0.05, *R*^2^ change = 0.218. The 95% confidence interval for the mediating effect of UDVA was (−71.825, −1.958), with zeros excluded from the upper and lower intervals, reflecting the existence of a mediating effect of UDVA in the influence of physical exercise on static equilibrium (eyes open). The ratio of the mediating effect of UDVA to the total effect was (0.432) × (0.287)/(0.411) × 100% = 30.17%.

A model test was conducted to examine the mediating effect of physical exercise with additional visual tasks on static balance. In the first step, the predictor variable was physical exercise with additional visual tasks (X), and the criterion variable was static balance (Y). A regression analysis was conducted between the two variables, and the result was a standardized regression coefficient A1 = 0.411, *p* < 0.05, *R*^2^ change = 0.159. In the second step, the predictor variable was physical exercise with additional visual tasks (X), and the criterion variable was UDVA (M). A regression analysis was conducted between the two variables, and the result was a standardized regression coefficient A2 = 0.432, *p* < 0.05, *R*^2^ change = 0.177. In the third step, the predictor variables were physical exercise with additional visual tasks (X) and UDVA (M), and the criterion variable was static balance (open eyes) (Y). A regression analysis was conducted between the three variables, and the result was a standardized regression coefficient A3 = 0.287, *p* < 0.05, *R*^2^ change = 0.218. By testing the mediating effect model of UDVA (M) (see Table 7), it was found that each step of the test was significant, proving that the mediating effect of UDVA was significant. The 95% confidence interval for the mediating effect of UDVA was (−71.825, −1.958), and the interval did not include zero, reflecting the mediating effect of UDVA in the relationship between physical exercise and static balance. The proportion of the total effect attributable to the mediating effect of UDVA was (0.432) × (0.287)/(0.411)*100% = 30.17%. The model diagram for the mediating effect is shown in Figure 1.

**Table 7:** The mediating effect of UDVA between physical exercise and static balance.

	Standardized regression equation	Regression coefficient test
1st step	Y = 0.411X	SE = 0.143, *t* = 4.135
2nd step	M = 0.432X	SE = 0.004, *t* = 4.393
3rd step	Y = 0.287 M	SE = 0.153, *t* = 2.697
	0.288X	SE = 4.013, *t* = 2.708

SE stands for standard deviation.

#### 3.3.5 Regression analysis of physical exercise, UDVA, and dynamic balance

Through the study, it was found that adding visual tasks to physical exercise had a significant effect on the dynamic balance of fourth-grade students. Therefore, a linear regression analysis was conducted between independent variables including physical exercise with additional visual tasks and UDVA (predictor variable), and dynamic balance as the dependent variable (criterion variable). From the regression analysis of physical exercise, UDVA, and dynamic balance, it showed *F* = 14.075, *p* = 0.000, R = 0.503, *R*^2^ variation = 0.235, indicating a significant difference in the regression equation. The standardized regression coefficient of UDVA on dynamic balance was 0.253, *p* < 0.05, with a significant difference.

**Figure 2:**
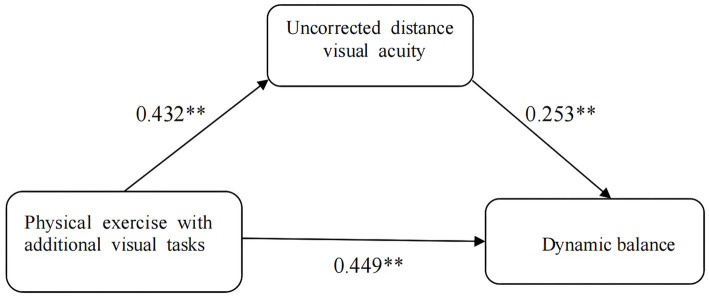
The mediating effect model of uncorrected distance visual acuity in physical exercise and dynamic balance. In the first step the independent variable was physical exercise with additional visual tasks (X), the dependent variable was dynamic balance (Y), and the regression analysis was performed between the two variables, which resulted in a standardized regression coefficient A1 = 0.449, *p* < 0.05, and *R*^2^ change = 0.192; in the second step the independent variable was physical exercise with additional visual tasks (X), and the dependent variable was UDVA (M), and the regression analysis was performed between the two variables. The result was a standardized regression coefficient A2 = 0.432, *p* < 0.05, *R*^2^ change = 0.177; the third step was a regression analysis between the independent variables of additional visual tasks of physical exercise (X) and UDVA (M), and the dependent variable was dynamic equilibrium (Y), and the result was a standardized regression coefficient A3 = 0.339, *p* < 0.05, *R*^2^ change = 0.235. The 95% confidence interval for the mediating effect of UDVA was (19.967, 78.058), with zeros excluded from the upper and lower intervals, reflecting the existence of a mediating effect of UDVA in the dynamic equilibrium of the influence of physical exercise. The ratio of the mediating effect of UDVA to the total effect was (0.432) × (0.339)/(0.449)×100% = 32.62%.

The mediating effect of physical exercise with added visual task on dynamic balance was tested through a model. In the first step, the independent variable was the physical exercise with added visual task (X), and the dependent variable was dynamic balance (Y). A regression analysis was conducted between the two variables, and the result was a standardized regression coefficient A1 = 0.449, *p* < 0.05, *R*^2^ variation = 0.192. In the second step, the independent variable was the physical exercise with additional visual task (X), and the dependent variable was UDVA (M). A regression analysis was conducted between the two variables, and the result was a standardized regression coefficient A2 = 0.432, *p* < 0.05, *R*^2^ variation = 0.177. In the third step, the independent variables were physical exercise with additional visual task of (X) and UDVA (M), and the dependent variable was dynamic balance (Y). A regression analysis was conducted between the three variables, and the result was a standardized regression coefficient A3 = 0.339, *p* < 0.05, *R*^2^ variation = 0.235. Through the mediating effect model test of UDVA (M) (see Table 8), it was found that each step of the test was significant, demonstrating that the mediating effect of UDVA was significant. The 95% confidence interval for the mediating effect of UDVA was (19.967, 78.058), and the upper and lower limits did not include zero, reflecting the mediating effect of UDVA on the relationship between physical exercise and dynamic balance. The proportion of the total effect accounted for by the mediating effect of UDVA is (0.432) × (0.339)/(0.449)×100% = 32.62%. The model diagram of the mediating effect is shown in Figure 2.

**Table 8:** The mediating effect of UDVA between physical exercise and dynamic balance.

	Standardized regression equation	Regression coefficient test
1st step	Y = 0.449X	SE = 0.109, *t* = 4.601
2nd step	M = 0.432X	SE = 0.004, *t* = 4.393
3rd step	Y = 0.339 M	SE = 0.118, *t* = 3.227
	0.253X	SE = 3.104, *t* = 2.403

SE stands for standard deviation.

## 4 Discussion

### 4.1 Current vision health development of 9–10 year-old children

In this study, the control group of students showed a downward trend in visual health, reflecting the severity of vision health problems among 9–10 year-old students. However, the downward trend in vision level of 9–10 year-old children may be related to the stages of visual development and prolonged use of near vision. Zhou et al. (25) found that the sensitive period for children’s KVA and dynamic visual acuity was between 6–8 years old. When children were in the 9–14 year-old stage, their vision begins to decline with age. The results of this experimental study that “the visual health of the subjects (9–10 years old) has significantly declined” corresponded to the above findings. Therefore, preventing and controlling poor vision requires attention to the developmental stage of vision, focusing on the sensitive period of visual development, and establishing a healthy concept of “preventing and controlling poor vision.” Epidemiological studies have found that prolonged use of near vision is an important factor causing children’s myopia. Zhu et al. (26) surveyed 1,803 students aged 9–11 years old and found that each myopic student used their eyes for close work for an average of 22 h per week. The prevention and control of myopia in students is mainly achieved through schools and families. So schools and parents should pay attention to children’s near vision problems, strictly control prolonged near vision study, emphasize the concept of “health first” and adjust visual usage time in a timely manner, achieving a balance between work and rest. Schools are the main body for preventing and controlling poor vision of children, and should focus on the healthy development of children’s physical and mental health, as well as build a practical system for myopia prevention and control. Schools and teachers can reasonably integrate visual training into physical exercise, so as to enhance the visual recognition function in a targeted manner, promote the body’s accurate and rapid recognition of environmental information, improve the body’s balance ability, and create a favorable internal environment for primary school students’ learning of motor skills. In addition, in physical education, the intervention training of additional visual switching tasks can be used to cultivate students’ awareness of distance-vision adjustment, exercise the regulation function of the ciliary muscle, improve students’ visual health, and promote students’ healthy physical and mental development.

### 4.2 Current balance ability development of 9–10 year-old children

This study found that the static and dynamic balance of the tested students (9–10 years old) showed an upward trend. Blodgett et al. (27) believed that the static and dynamic balance abilities of elementary school students aged 7–10 years old would improve with age. This was consistent with the result of this study that “students’ static and dynamic balance in this age group (9–10 years old) showed a significant upward trend.” The childhood stage is an important period for developing balance ability, and 8–10 years old is a sensitive period for balance ability (28). At this stage, children and adolescents develop rapidly both physically and mentally, making it a sensitive period for physical fitness development. In this case, effective exercise training should be carried out to enhance physical fitness and develop bodily functions. Therefore, 9–10 years old is an key stage for developing children’s balance ability, and should be seized to carry out efficient physical exercise, improve children’s physical balance ability, and lay a foundation for future sports skill learning.

### 4.3 Analysis of the effect of physical exercise with additional visual tasks on children’s vision and balance

This study found that after 16 weeks of visual task added to physical exercise, the experimental group of students (9–10 years old) showed a significant improvement in KVA and UDVA, while the control group showed a significant decrease in these measures. Thus, adding visual tasks to physical exercise had a significant promoting effect on children’s vision. Cao et al. (29) and others found that additional visual task to physical exercise had a positive effect on improving the vision of 6–9 year old students, and could be used as a training method to improve their vision health. This was consistent with the results of this study, which showed that “students significantly improved their vision through the additional visual tasks to physical exercise.” This may be because physical exercise with visual task improved the adjusting ability of the ciliary muscle through near-far visual regulation training, thus improving students’ KVA. The trends in children’s KVA and UDVA changed with age, and the two were positively correlated. Improving KVA can lead to an improvement in UDVA.

As the ciliary muscle is a smooth muscle that is innervated by both sympathetic and parasympathetic nerves and has structural features of fast striated muscle, such as nerve conduction, histological composition, and microstructure. Additionally, the ciliary muscle contains abundant endoplasmic reticulum and mitochondria, especially in the radial and circular fibers (30). Therefore, the ciliary muscle can be strengthened through training. Studies on using physical exercise as a carrier for ciliary-muscle regulation training to improve visual health have increased. Wylȩgała (31) found that adding dynamic visual tasks to physical exercise could improve eye physiological functions, reduce eye pressure, enhance choroidal blood circulation, and ensure adequate blood supply to the retinas. Yin et al. (32) and others believed that regular physical exercise could effectively prevent and control students’ myopia, and had a significant effect on improving KVA, and the core mechanism was mainly to improve the regulating sensitivity of the crystalline lens, ciliary zonules, and ciliary muscles through near-far visual training during exercise. Thus, additional visual tasks in physical exercise can replace ciliary-muscle regulation training, promote full relaxation of the ciliary muscle, and reduce ciliary muscle spasms, which improved children’s visual sensitivity and their visual health.

This study showed that through 16 weeks of visual task training on tested students (9–10 years old), it was found that both static and dynamic balance significantly improved in all groups, and the experimental group showed a more significant improvement in balance than the control group. It can be seen that visual exercise had a significant promoting effect on children’s static and dynamic balance when performed under open-eye conditions. This result may be due to the fact that the visual task mainly adjusted the ciliary muscle by alternating between far and near vision, which allowed for some visual improvement and led to a significant improvement in balance ability. Alghadir et al. (33) and other scholars have pointed out that visual situation significantly affected the balance ability of teenagers, and loss of visual input could significantly decrease their balance ability during movement. Schmidt et al. (34) have found that after visual deprivation in normal individuals, the frequency of the gravity center oscillation in double-legged support reduced, the amplitude was larger, and the stability of the body was significantly decreased. This was consistent with the results of this study that “tested students significantly improved their balance ability through visual training.” Therefore, as 7–12 year old are a critical stage for developing balance ability, targeted training should be provided to enhance their balance ability and promote their overall improvement of physical fitness.

### 4.4 Relationship between children’s vision and balance ability

The results of this study showed that there was a certain correlation between the experiment group students’ UDVA, KVA, static balance, and dynamic balance. Specifically, there was a significant positive correlation between the experiment group’s KVA and UDVA (*p* < 0.01). Freeman et al. (16) found that children with stronger KVA had clearer observation ability in the external environment in daily learning, and there was a certain correlation between KVA and UDVA. Sun et al. (35) also found that there was a positive correlation between children’s KVA and UDVA, and enhancing KVA was beneficial to improving students’ UDVA. These results were consistent with the findings of this study that “the KVA and UDVA of the tested students were positively correlated after additional visual tasks training in physical exercise.”

Thompson et al. (36) pointed out that students with visual impairments would affect the development of balance ability, especially for poor vision students. The results of this study confirmed this view, as UDVA showed a positive correlation with both static balance and dynamic balance in the experiment group students (*p* < 0.01 for both). This may be because improving visual acuity enabled students to accurately evaluate external information, quickly respond, automatically adjust posture, and maintain body balance by improving their spatial positioning, movement reaction speed, and movement accuracy.

### 4.5 Mediating effect of visual acuity on children’s balance ability in physical exercise

The results of this study indicated that UDVA partially mediated the effect of physical exercise with visual task on the balance ability of fourth-grade students. Specifically, there was a trend for fourth-grade students’ balance ability to change with the effect of additional visual task in physical exercise on UDVA, validating the research hypothesis. Onofrei and Amaricai (24) suggested that physical exercise and visual acuity were important factors that affected human postural balance, and that physical balance was influenced by the interaction of physical exercise and visual acuity. In this study, the partial mediating effect of UDVA on the impact of physical exercise on balance ability confirmed this view.

Taneda et al. (37) found that temporary visual impairment significantly reduced normal individuals’ balance ability, and a decrease in peripheral visual information further lowered body balance. Mani et al. (38) pointed out that the visual system played an important role in self-motion perception and spatial cognition, and visual stimuli from far and near vision could affect postural control during static standing. Strang et al. (18) argued that restricted vision limited the ability to adjust posture and balance during movement, increasing body sway and area and weakening the coordination of rotation, thus affecting dynamic balance ability. It was evident that UDVA was an important factor in maintaining body balance, and improving it can enable individuals to quickly and accurately identify external information and react, creating a favorable internal environment for maintaining body balance and significantly improving balance ability. In conclusion, visual acuity is a crucial factor in maintaining body balance, and improving UDVA can provide better visual stimulation, so as to better coordinate our body to maintain balance, and promote the development of balance.

## 5 Conclusion

Sports exercises with additional visual tasks had a positive effect on improving children’s visual health and balance ability. There was a positive correlation between children’s KVA and UDVA, and UDVA was positively correlated with both static and dynamic balance. UDVA had a partial mediating effect on the impact of sports exercises on children’s balance ability.

## 6 Shortcomings and prospects

The effects of physical exercise with additional visual tasks on students’ visual acuity and balance ability were explored experimentally by taking physical exercise with additional visual tasks as the experimental group and ordinary physical exercise as the control group. However, the different effects of physical exercise with other additional tasks on students’ visual acuity and balance compared with physical exercise with additional visual tasks need to be further explored and investigated.

## Data availability statement

The original contributions presented in the study are included in the article/supplementary material, further inquiries can be directed to the corresponding authors.

## Ethics statement

The studies involving human participants were reviewed and approved by the Ethics Committee of Suzhou University. Written informed consent to participate in this study was provided by the participants’ legal guardian/next of kin. The studies were conducted in accordance with the local legislation and institutional requirements. Written informed consent for participation in this study was provided by the participants’ legal guardians/next of kin.

## Author contributions

RY: Conceptualization, Formal analysis, Funding acquisition, Investigation, Methodology, Resources, Supervision, Validation, Writing – original draft. GZ: Data curation, Writing – original draft. AL: Conceptualization, Formal analysis, Investigation, Validation, Visualization, Writing – original draft. MW: Data curation, Writing – review & editing. LL: Project administration, Writing – review & editing. SD: Project administration, Writing – review & editing.
